# Evaluation of the acoustic performance of a novelty cathodic protection system against guitar steel strings corrosion

**DOI:** 10.1016/j.heliyon.2024.e30811

**Published:** 2024-05-08

**Authors:** Jorge Segura Alcaraz, José Antonio Bonastre Cano, Ernesto Juliá Sanchis, José María Gadea Borrell, Francisco Javier Cases Iborra

**Affiliations:** Universitat Politècnica de València, Campus of Alcoy, Pl. Ferrándiz y Carbonell s/n, 03801, Alcoy, Spain

**Keywords:** Acoustics, Guitar steel strings, Cathodic protection, Corrosion

## Abstract

This work was conducted to establish the efficiency of an impressed current cathodic protection system for musical instruments’ steel strings in protecting them from corrosion caused by human sweat. To conduct this research, the harmonic content degradation of a guitar string subjected to different corrosion stages by artificial human sweat, with and without cathodic protection by an impressed current, was studied. String corrosion is characterised by not only the electrochemical technique of polarisation resistance, but also by weight loss by gravimetric measurements and FESEM microscopy. From the correlation between the acoustic and electrochemical results, it can be concluded that harmonic content degradation of guitar strings increases corrosion but is less significant in the strings protected by impressed current.

## 1 Introduction

Several research works are related to the sound of strings in musical instruments. Studies are generally based on analysing the harmonic spectrum of the string signal using Fourier Transform or Power Spectral Density (PSD) [[1], [2], [3], [4], [5]] combined with numerical and analytical studies [5,6].

Very few studies have focused on investigating the change in "string sound" due to the corrosive action of sweat on performers’ hands. This action caused by human sweat, which can lead to allergic skin problems like atopic dermatitis [7], has been studied by creating artificial human sweat and electrochemical techniques for measuring corrosion [[8], [9], [10], [11]]. The first studies into the correlation between harmonic spectrum degradation and string corrosion by artificial human sweat were performed by the authors in a previous research work [12]. By taking into account works on the material and structure of metal guitar strings [13], the authors propose an impressed current cathodic protection system that considerably reduces physical deterioration [14].

The present work studies the effectiveness of a cathodic protection system in avoiding the acoustic harmonic content degradation of guitar strings by the action of human sweat. These changes in strings' harmonic content due to corrosion affect string performance and produce variations in the instrument's sound. The system is validated by correlating electrochemical and acoustic results for a new string and a string subjected to a 96-h corrosion process. The results show that the cathodically protected strings present less harmonic sound spectrum degradation and less physical weakening due to the corrosive action of artificial sweat. The obtained results do not allow us to suggest the construction of a guitar prototype that includes this protection against corrosion, which would prolong strings' life span.

## 2 Materials and methods

### 2.1 Strings

The guitar strings used in the experimental process are D'Addario D4 NW026 (diameter of 0.61 mm). Strings are made up of two parts: the first is the steel core with a hexagonal cross-section; the second is a metal wrap wire. The core is mechanically resistant and supports the axial stress of a string subjected to the tension of its tuning for a fundamental vibration mode of 147 Hz. Its chemical composition, obtained by an EDX analysis with steel standards and expressed as a mass percentage (%), is Fe 98.77, Mn 0.52, C 0.41 and Si 0.30.

The metal wrap wire, or roundwound, is made of carbon steel and its cross-section is circular. The roundwound has a nickel microcoating and is twisted around the core of the string. The chemical composition of the roundwound core, obtained by an EDX analysis with steel standards expressed as a mass percentage (%), is Fe 73.95, Si 15.78 and C 10.56.

### 2.2 Artificial human sweat

Strings were subjected to a controlled corrosion process using solutions that simulated the electrolytic composition of human sweat in accordance with Standards UNE-EN 1811:1999+A1 and EN ISO 17700. The chemical composition of artificial human sweat, expressed mass percentage dissolved in water, is as follows: sodium chloride 0.5 %, lactic acid 0.1 % and urea 0.1 %. Ammonia 1 % was added until a pH of 6.5 ± 0.1 was reached.

### 2.3 Electrochemical, weight loss and FESEM measurements

Strings were soaked with this artificial sweat solution, and subjected to a corrosion process that involved exposure lasting 24 h, 48 h, 72 h and 96 h. For each corrosion stage, the following experimental procedures were performed.

- Estimating strings' polarisation resistance. Ends of strings were connected by clamps to an Ecochemie Autolab PGSTAT302 potentiostat-galvanostat. Rp measurements were taken at a scan rate of 1 mVs−1. The potential scan was carried out from −10 mV to +10 mV in relation to the open circuit potential (OCP). Tafel curves were measured at a scan rate of 1 mVs−1 (±250 mV vs. OCP).
- Taking gravimetric measurements at the end of each experimental stage to quantify the weight loss caused by corrosion.
- Studying samples' morphology to search for oxides using a FESEM Ultra Zeiss 55 by applying an acceleration voltage of 3 kV. Energy dispersive X-ray (EDX) measurements were taken between 0 and 20 kV.

The description of the measurement process for the electrochemical tests, gravimetric weight loss measurements and FESEM microscopy, as well as the obtained results, have been previously described in detail by the authors [14].

### 2.4 Impressed current protection

The following ionic conducting electrolyte for cathodic protection was used: 0.1 M potassium nitrate, 99 % from Panreac (Reag.Ph.Eur).

Two BLAUSONIC 0–30V 2.5A direct current power supplies provided the potential and intensity of the electrical current needed for impressed current cathodic protection. To measure the values of the potential difference and the current at the different circuit points during the experimental process, multimeters PROINSA 3511927 and GOLDSTAR DM-311 were employed. By means of an Ag/AgCl (3 M) reference electrode, which came into contact with the conductive electrolyte, several electrical control measurements were taken.

**Fig. 1:**
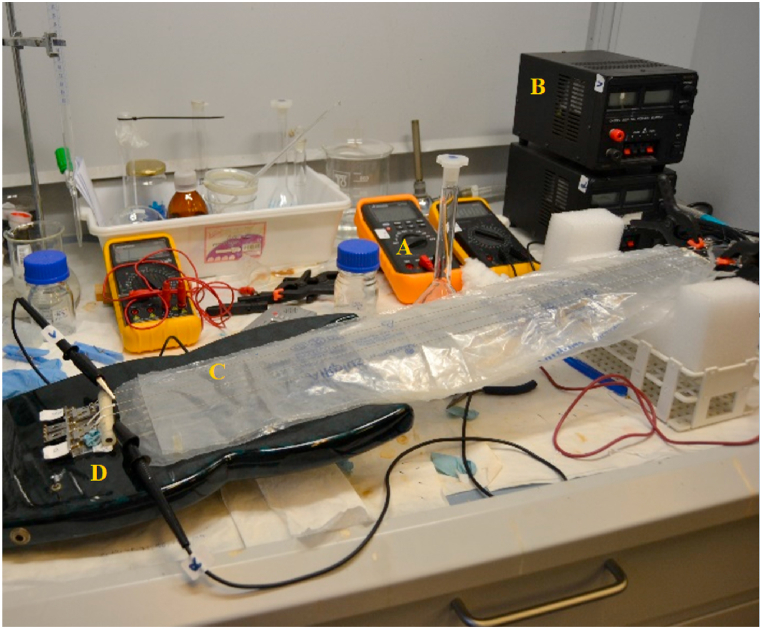
Experimental setup for impressed current cathodic protection. A) Multimeter; B) Power supply; C) Protected strings; D) Guitar.

**Fig. 2:**
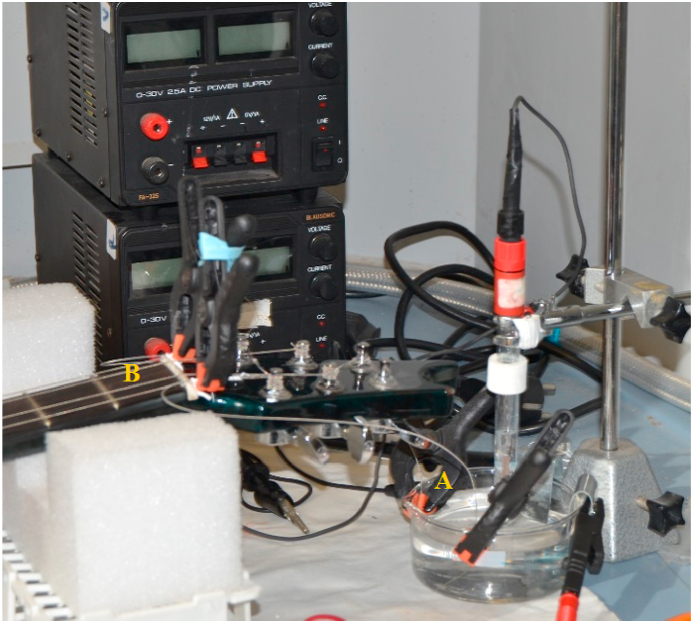
String immersed in 0.1 M KNO_3_ for impressed current cathodic protection. A) Electrolyte for cathodic protection; B) Protected strings.

To simulate real working conditions, strings were kept in pitch at their fundamental frequency of 147 Hz and an axial force of 89 N using a KORG GA-30 tuner throughout the corrosive process. Fig. 1, Fig. 2 show the power supplies and the experimental setup with the guitar.

### 2.5 Acoustic measurements

**Fig. 3:**
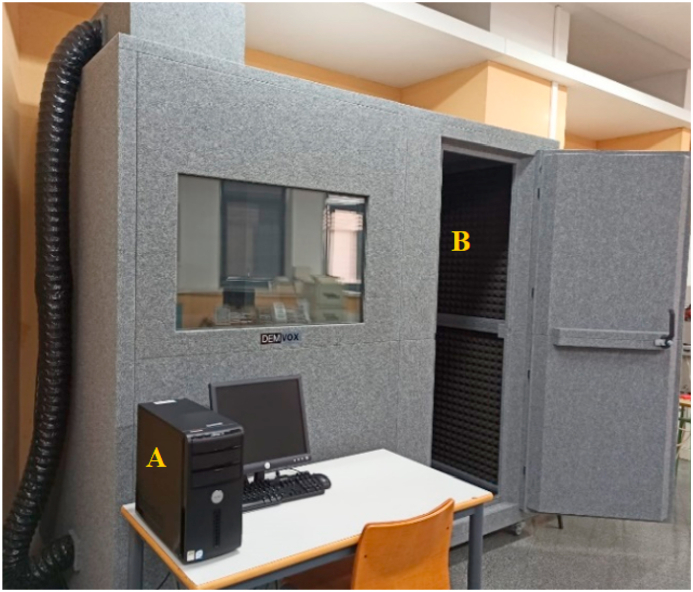
Semi-anechoic chamber. A) PC for data acquisition and analysis; B) Semi-anechoic chamber.

To take acoustic measurements in each corrosion process stage, a B&K 4189-A-021 microphone and a preamplifier B&K model 2671, connected to a NI USB 9234 data acquisition card at a sampling rate of 10000 Hz, were used. The signal was obtained by the Data Acquisition Matlab Toolbox. The experimental setup was placed in a DEMVOX semi-anechoic chamber to acoustically isolate the system from the outside so that the signal would not contain any measurement room frequencies. According to Standard ISO 717-1, the chamber can be used within the frequency range from 100 to 3500 Hz. Fig. 3 shows the semi-anechoic chamber employed for the acoustic measurements.

Three amplitude-time signals were recorded by plucking the string and the average was calculated. The Matlab Signal Processing Toolbox was used to calculate the Fast Fourier Transform (FFT), the Power Spectral Density (PSD) with Equation (1) and Equation (2), respectively, and the spectrogram:

(1) $$FFT\left(x_{j}\right)=\sum_{j=0}^{n-1}\omega^{jk}x_{j+1}$$

where *x*_*j*_ is the signal and $\omega =e^{‐2\pi i/n}$.

(2) $$PSD\left(x\right)=\frac{1}{\left(f_{s}\cdot N\right)}\cdot {\left|FFT\left(x_{j}\right)\right|}^{2}$$

where *f*_*s*_ is sampling frequency.

The FFT of the signal is used to calculate loss factor by the Half Power Method [15].

## 3 Results and discussion

The string signals analysis showed that the harmonic string content changed as corrosion progressed. Table 1 provides the values of strings polarisation resistance of the string (Rp), mass loss, the total PSD, the quotient in the PSD percentage of the fundamental frequency in relation to the total PSD, and fundamental frequency loss factor. Table 1 includes the electrochemical and acoustic results for the new strings, and for the cathodic protected and unprotected strings after 96 h of corrosion.

**Table 1:** Correlation between the electrochemical and acoustic results.

String	Rp (Ω·cm^2^)	Mass Loss (g)	Total PSD (Pa^2^/Hz)	PSD fundamental frequency at 147 Hz (Pa^2^/Hz)	PSD fundamental frequency/PSD total (%)	Fundamental frequency loss factor at 147 Hz
New		–	2.9927·10^−4^	7.3930·10^−5^	24.70	0.0015
Protected (96 h)	2402	0.0153 (0.6 %)	6.4774·10^−5^	1.8044·10^−5^	27.85	0.0041
Unprotected (96 h)	668	0.0245 (1 %)	2.2467·10^−5^	7.7998·10^−6^	34.71	0.0137

Loss factor for the fundamental mode increased with the oxidation process from the original of 0.0015–0.0041 with the protected string and up to 0.0137 with the unprotected string. This rise in loss factor in the unprotected string resulted in greater kinetic energy loss in the string as it vibrated.

The PSD of the acoustic signal, which is related to the string's acoustic energy when plucked, decreased after 96 h of corrosion from 2.9927·10^−4^ (Pa^2^/Hz) for the new string to 6.4774·10^−5^ (Pa^2^/Hz) for the protected string and, finally, with 2.2467·10^−5^ (Pa^2^/Hz) for the unprotected string. The decrease in PSD during corrosion confirmed the string's loss of kinetic energy as it vibrated, especially the unprotected string, due to the increased frictional damping caused by corrosion oxide.

As corrosion progressed, loss of signal harmonics occurred with a rise in the ratio between the PSD of the fundamental frequency at 147 Hz and the total PSD of the signal. The PSD of the fundamental frequency/total PSD ratio of the new string was 24.70 %. After 96 h of corrosion, the PSD of the fundamental frequency/total PSD ratio was 27.85 % for the protected string and 34.71 % Hz for the unprotected string. These results indicate that corrosion produced continuous acoustic signal degradation, which affected mainly higher harmonics, and left the fundamental harmonics of 147 Hz more present in the signal.

**Fig. 4:**
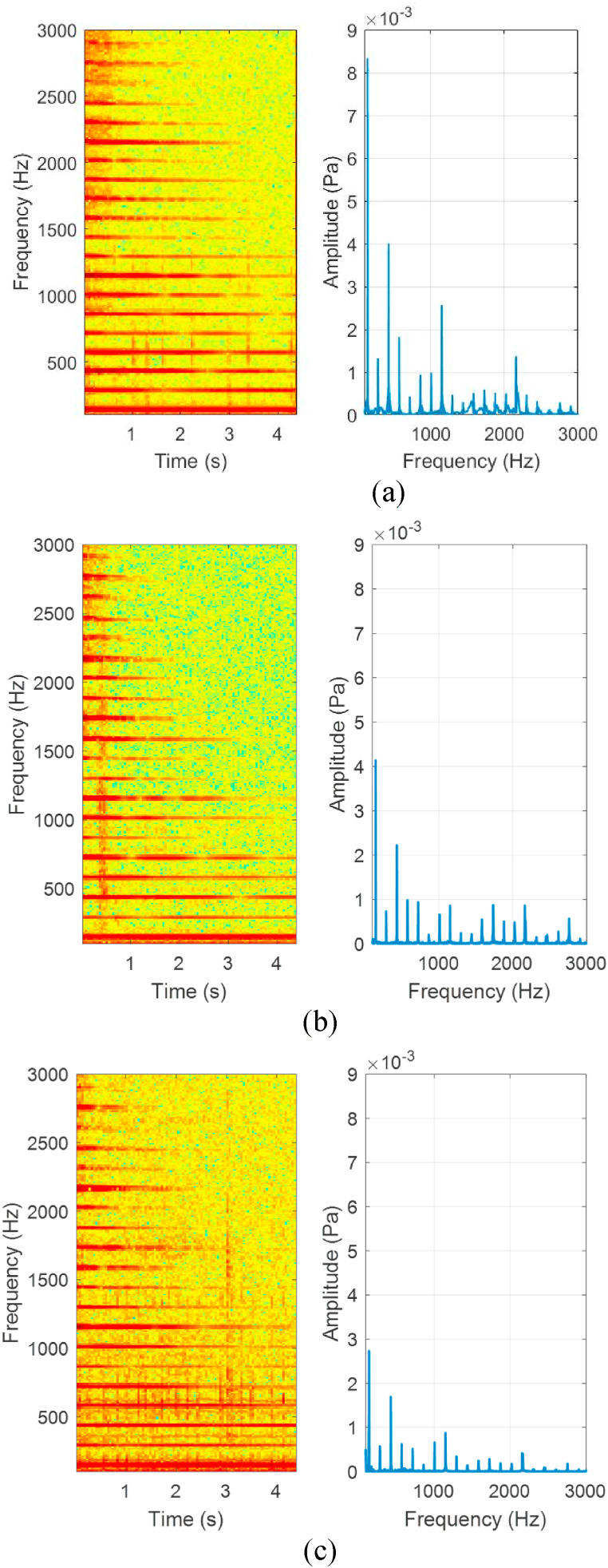
Spectrograms and FFTs of (a) New string. (b) Protected string after 96 h corrosion. (c) Unprotected string after 96 h corrosion.

The spectrogram and FFT analysis also indicated loss of signal harmonics with corrosion. After 96 h of corrosion however, the cathodically protected string had similar harmonic content to that of the new string. Nevertheless, the unprotected string displayed significant harmonics loss in amplitude and duration over time, especially as of 2000 Hz. Fig. 4 (a), (b) and (c) shows the spectrogram and the FFT of the acoustic signals.

**Fig. 5:**
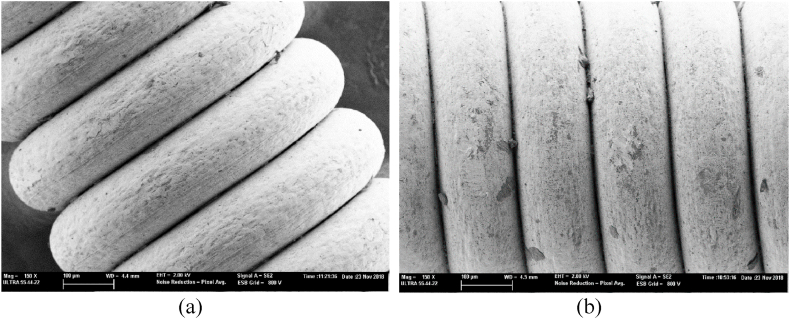
FESEM microscopy images of (a) Cathodic protected string. (b) Unprotected string.

Acoustic data correlated directly with the electrochemical measurements and mass loss values. Cathodic protection slowed down the corrosion process by maintaining the acoustic properties of new strings for longer periods. After 96 h of corrosion, the strings with cathodic protection showed 400 % higher polarisation resistance and 60 % lower mass loss. Both findings indicate less corrosion. FESEM microscopy validated the electrochemical and acoustic results. In a new string, the roundwound was grey and no oxide growth occurred. Oxides had formed in the interstitial roundwound zones after the 96-h corrosion period. These oxides were shown as dark areas. In the protected string, there were fewer dark areas where corrosion effects had formed. Fig. 5 shows the FESEM microscopy images of: a) the protected string; b) the unprotected string after corrosion.

## 4 Conclusions

This work presents a solution to protect the steel strings of musical instruments against corrosion caused by human sweat by means of impressed current cathodic protection. The electrical currents and voltages herein used for cathodic protection make it feasible to implement the system in commercial instruments using batteries with low voltage and electrolyte capsules that occupy very little space and small volumes.

The strings with the proposed cathodic protection show more polarisation resistance, lower mass loss and fewer formed oxides, which all imply less corrosion. The electrochemical and acoustic results correlate directly with corrosion progress. The corrosion process causes loss of kinetic energy in the string as it vibrates, especially in higher harmonics, with the presence of fundamental harmonics dominating sound. It can be stated that adopting a cathodic protection system for strings allows them to preserve their acoustic properties for longer periods, avoids them failing and prolongs strings’ life span.

To prevent corrosion problems, this cathodic protection system can be extrapolated to any other type of steel cable structure. This method would be particularly applicable to any other orchestral instrument with metal strings that have a cross-section with a core and wrap wire.

## Data availability statement

Data will be made available on request.

## CRediT authorship contribution statement

**Jorge Segura Alcaraz:** Writing – review & editing, Writing – original draft, Software, Methodology, Investigation. **José Antonio Bonastre Cano:** Writing – review & editing, Resources, Methodology, Investigation. **Ernesto Juliá Sanchis:** Writing – review & editing, Supervision, Methodology, Investigation. **José María Gadea Borrell:** Writing – review & editing, Methodology, Investigation. **Francisco Javier Cases Iborra:** Writing – review & editing, Validation, Methodology, Investigation.

## Declaration of competing interest

The authors declare that they have no known competing financial interests or personal relationships that could have appeared to influence the work reported in this paper.
